# Prevalence of post-traumatic stress disorder among residents of Shanghai standardized training programs during the COVID-19 outbreak: a cross-sectional study

**DOI:** 10.3389/fpubh.2023.1203333

**Published:** 2023-10-05

**Authors:** Ruiwen Huang, Chao Tang, Jianfeng Luo, Tingting Li, Li Wang, Chang Li, Lu Cao, Shiyu Wu

**Affiliations:** ^1^Department of Science and Education, RuiJin Hospital LuWan Branch, School of Medicine, Shanghai Jiaotong University, Shanghai, China; ^2^Department of Psychiatry, Xuhui Mental Health Center, Shanghai, China; ^3^Department of Biostatistics, School of Public Health, Fudan University, Shanghai, China; ^4^NHC Key Laboratory of Health Technology Assessment, Fudan University, Shanghai, China; ^5^Key Laboratory of Public Health Safety of Ministry of Education, Fudan University, Shanghai, China; ^6^Nursing Department, RuiJin Hospital LuWan Branch, School of Medicine, Shanghai Jiaotong University, Shanghai, China; ^7^Graduate Medical Education Office, Shanghai Pudong New Area Gongli Hospital, Shanghai, China; ^8^Teaching Affairs Office, Shanghai Seventh People's Hospital, Shanghai, China; ^9^Department of Orthopedic Surgery, Zhongshan Hospital, Fudan University, Shanghai, China

**Keywords:** post-traumatic stress disorder, COVID-19, residents, standardized residency training program, perceived social support

## Abstract

**Background:**

The COVID-19 pandemic may have increased the prevalence of psychiatric disorders, such as anxiety, depressive disorders, and post-traumatic stress disorder (PTSD), among healthcare workers.

**Purpose:**

This study aims to investigate the prevalence of PTSD and its risk factors among residents in the standardized residency training programs (SRTPs) in Shanghai during the COVID-19 outbreak.

**Participants and methods:**

An online cross-sectional survey was conducted between December 17, 2021, and January 7, 2022, among SRPT residents from 15 hospitals in Shanghai, China. Questionnaires comprising general information, medical-related traumatic event experiences, the PTSD Checklist (PCL-5), and the perceived social support scale (PSSS) were distributed to the participants using the online Questionnaire Star electronic system.

**Results:**

We included 835 valid responses for the analysis. In total, 654 residents (78.3%) had experienced at least one traumatic event, and 278 residents (33.3%) were found to have PTSD symptoms. The age 26–30 years old, female sex, and increased resident working hours were identified as the risk factors for PTSD (*p* < 0.05), and perceived social support had a significant negative association with PTSD (*p* < 0.05).

**Conclusion:**

During the COVID-19 pandemic, there was a high prevalence of PTSD among SRTPs residents in Shanghai. The age 26–30 years old, female sex, and increased resident working hours were identified as risk factors for PTSD, while perceived social support was identified as a protective factor against PTSD. The present findings can be applied in STRPs management and provide useful information for designing special interventions and protocols for SRTPs residents.

## 1. Introduction

On 11 March 2020, the World Health Organization (WHO) reported severe acute respiratory syndrome-coronavirus-2 (SARS-CoV-2) as the causative agent of coronavirus disease-2019 (COVID-19) and declared the pandemic status of COVID-19 (1). The pandemic prompted an assertive public health response worldwide, including social isolation as well as the closure of schools, businesses, and other establishments, all of which contributed to a rise in various mental health problems among citizens (2). Healthcare workers (HCWs) working at the frontline of this pandemic experienced high stress levels and were particularly at risk for persistent mental health problems, such as depression, chronic psychological stress, anxiety, insomnia, and posttraumatic stress disorder (PTSD) (2). Importantly, these mental health problems, in turn, could result in hazards exceeding the consequences of the COVID-19 pandemic itself (3).

PTSD is a significant economic burden while a highly prevalent condition. The prevalence of probable PTSD in HCWs is about three times than observed in the general population. This pattern is due to the long-term work-related stressors HCWs must endure (4). Studies have suggested a high risk for PTSD development among HCWs who have been involved in the three major recent coronavirus disease-related outbreaks: severe acute respiratory syndrome (SARS), Middle East respiratory syndrome (MERS), and COVID-19 (5). More specifically, it has consistently been shown that a high proportion of HCWs is at a high risk of developing PTSD due to the COVID-19 pandemic (6, 7), and the individual-level risk factors for this phenomenon include pre-existing mental-health concerns (8), being in the nursing profession (9), being female (10), and young age (11).

In China, standardized residency training programs (SRTPs) were first implemented in Shanghai in 2010 to set quality standards for residency training (12). Since then, all hospitals in Shanghai have hired physicians only with certifications from SRTPs. SRTPs are considered an important resource for continuing education and receiving system-based clinical training (13). In general, the importance of SRTPs has been demonstrated because they provide residents with more standardized training and ensure that the residents are highly qualified. However, the transition to a SRTP leads to challenges, such as the uncertainty of employment and less personal attachment to the workplace for SRTP residents. Moreover, entering an SRTP adds 3 years of highly stressful training and relatively low-paid work for young physicians (14). Given the work-related stressors, pressures elicited by patients, and challenging daily work routines, SRTP residents may be at an increased risk of psychopathological stress-related disorders. Zhang and colleagues reported that the prevalence of depression disorder was 28.3% among SRTP residents (15). Thus, it would not be surprising if the prevalence of PTSD among SRTP residents was much higher than that in the general population.

Studies have found that young age, low work experience, heavy workload, working in unsafe settings, as well as a lack of training and social support are predictors of stress-related symptoms (16). SRTP residents are more likely than other HCWs to experience traumatic events and develop PTSD due to their high-pressure roles, and this is especially pertinent in relation to the COVID-19 pandemic. Therefore, given that SRTP residents are one of the populations most vulnerable to PTSD among HCWs, we investigated the prevalence of PTSD and its risk factors among SRTP residents during the COVID-19 outbreak from 2019 to 2021.

## 2. Materials and methods

### 2.1. Study design and participants

The Department of Ethics Commission of Ruijin Hospital/Lu Wan Branch within the School of Medicine of Shanghai Jiaotong University (Shanghai, China) provided ethical approval (LWEC2020031) for our study protocol.

Online informed consent for participation was obtained upon completion of the questionnaire. Fifteen hospitals providing SRTPs in Shanghai were selected as research units using a multistage method based on stratified random-cluster sampling. The online Questionnaire Star electronic system (www.wjx.cn/) was used, and questionnaires were distributed to participants as a link via the WeChat (www.wechat.com/), with the help of the education departments of selected hospitals. The inclusion criterion for participants was residents enrolled in an SRTP from 2019 to 2021.

### 2.2. Screening questionnaire

The screening questionnaire was written in Chinese and comprised four main components: general information, medical-related traumatic experiences, PTSD Checklist for the *Diagnostic and Statistical Manual of Mental Disorders, Fifth Edition (DSM-5, PCL-5)*, and the perceived social support scale (PSSS).

#### 2.2.1. General information

The general data of participants collected were hospital level, sex, age, education, medical specialty, training grade, marital status, average number of hours worked per week, and salary.

#### 2.2.2. Medical-related traumatic events

The checklist for medical-related traumatic events in the questionnaire comprised nine common traumatic events: “criticism/bullying by senior doctors,” “criticism/bullying by other SRTP residents,” “failing an important examination,” “iatrogenic exposure/infection,” “conflict with patients/family members, being threatened or attacked,” “medical negligence and malpractice,” “patients died/deteriorated,” “medical isolation/separation,” and “yourself or hospital colleague fell seriously ill.” If participants had experienced traumatic events that were not included in this checklist, they could describe it as “other.” Furthermore, those who reported at least one traumatic experience were required to select the most affecting traumatic event and continue to PCL-5 and the PSSS. If they had not suffered a traumatic experience, the survey was terminated, and participants were excluded from the diagnosis of PTSD.

#### 2.2.3. PCL-5

PCL-5 is a self-reported screening measurement with 20 items to assess PTSD symptoms. The responses on PCL-5 are given using a Likert scale, and the severity of each symptom is divided into five levels: 0 = “not at all”; 1 = “a little bit”; 2 = “moderately,” 3 = “quite a bit”; 3 = “extremely.” The maximum score is 80. We used a cutoff score of 33 points (7) to determine a diagnosis of PTSD, and this scale had satisfactory reliability and validity (Cronbach’s α = 0.94).

#### 2.2.4. PSSS

The PSSS consists of 12 items, with four items measuring “family support,” four items measuring “friend support,” and four items measuring “significant other support.” The responses for each item are ranked on a seven-point Likert scale (1 = “strongly disagree”; 7 = “strongly agree”). The total score of perceived social support is the sum of all items, with a high score indicating a high level of perceived support.

In the present study, social support was categorized into three levels according to the total score: “low” (PSSS score < 37), “moderate” (37 ≤ PSSS score ≤ 60), and “high” (PSSS score > 60) (17). The Chinese version of the PSSS was found to have adequate internal consistency (Cronbach’s α = 0.94) among undergraduate students during the early phases of the COVID-19 pandemic (18). In the present study, Cronbach’s alpha of the total scale was 0.93.

### 2.3. Data collection

**Figure 1:**
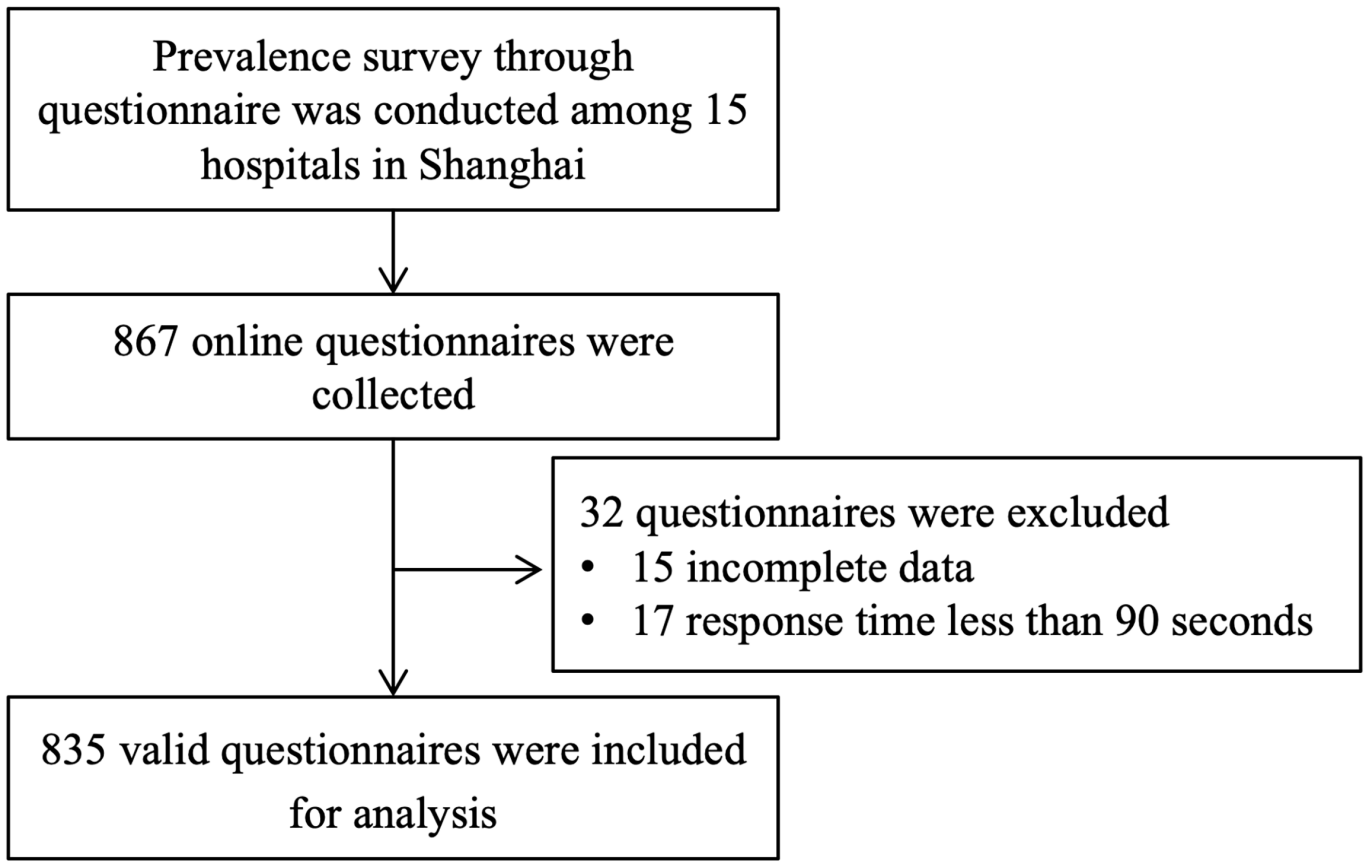
Flowchart of the study.

A total of 867 online questionnaires from 15 hospitals were collected, and 32 questionnaires were excluded because of incomplete data (15 questionnaires) or response times of less than 90 s (17 questionnaires). Finally, 835 valid responses were included valid for analysis, the response rate was 96.3%, as shown in Figure 1.

### 2.4. Data analysis

Statistical analyses were undertaken using SPSS 25.0 (IBM, Armonk, NY, United States). Categorical variables are expressed as absolute values (percentages). The internal consistency of the total scores of PCL-5 and the PSSS was evaluated using Cronbach’s alpha. Analyses of descriptive statistics were conducted to examine the demographic and other selected characteristics of participants. Pearson’s chi-square test or Fisher’s exact test were used to compare differences among subgroups. Multivariate logistic regression was employed to assess the risk factors for PTSD variables that were significant in the univariate analysis at *p* ≤ 0.10. *p* < 0.05 was considered significant unless stated otherwise.

## 3. Results

Table 1 summarizes the sociodemographic characteristics and PTSD prevalence of respondents. Most (92.7%) respondents were from a third-class hospital. Most respondents were male (*n* = 493, 59.0%). The prevalence of PTSD was higher in women (41.5%) than in men (27.6%). Respondents were between 20 and 30 years of age (*n* = 776). Respondents were mainly undergraduates (*n* = 445, 53.3%). Specifically, 194 respondents were in their third year, 266 were in their second year, and the largest number of respondents were in their first year (*n* = 373, 44.7%) of training. Most respondents: were medical practitioners (*n* = 608, 72.8%); were unmarried (*n* = 710, 85.0%); worked 50–60 h per week (*n* = 340, 40.7%); had a monthly salary of 5,001–8,000 yuan (*n* = 465, 55.7%).

**Table 1:** Baseline characteristics of study participants (*N* = 835).

Characteristic	*N*	%	PTSD + *N* (%)	*p*
*Types of medical institutions*				
Third class hospital	774	92.7%	264 (34.1%)	0.121
Second class hospital	57	6.8%	16 (28.0%)	
Other	4	0.5%	1 (25.0%)	
*Sex*				
Female	342	41.0%	142 (41.5%)	0.000*
Male	493	59.0%	136 (27.6%)	
*Age*				
20–25	362	43.4%	109 (30.1%)	0.321
26–30	414	49.6%	148 (35.7%)	
>30	59	7.1%	21 (35.6%)	
*Education*				0.986
Undergraduate	445	53.3%	147 (33.0%)	
Graduate	247	29.6%	83 (33.6%)	
Doctor	143	17.1%	48 (33.6%)	
*Grade*				0.735
Third year	194	23.2%	63 (32.5%)	
Second year	266	31.9%	92 (34.6%)	
First year	373	44.7%	123 (33.0%)	
Postpone graduation	2	0.2%	0 (0.0%)	
*Medical practitioner or not*				0.357
Yes	608	72.8%	208 (34.2%)	
No	227	27.2%	70 (30.8%)	
*Marital status*				0.295
Single	710	85.0%	239 (33.7%)	
Married	124	14.9%	38 (30.6%)	
Other	1	0.1%	1 (100.0%)	
*Average hours work per week*				0.000*
<50 h	188	22.5%	39(20.7%)	
50–60 h	340	40.7%	108 (31.8%)	
60–70 h	173	20.7%	72 (41.6%)	
70–80 h	61	7.3%	22 (36.1%)	
>80 h	73	8.7%	37 (50.7%)	
Monthly salary (CNY)				0.464
<5,000	280	33.5%	99 (35.4%)	
5,001–8,000	465	55.7%	154 (33.1%)	
8,001–10,000	66	7.9%	21 (31.8%)	
10,001–15,000	18	2.2%	3(16.7%)	
>15,000	6	0.7%	1 (16.7%)	
*Social support*				0.000*
Low (<37score)	50	6.0%	31 (62.0%)	
Median (37–60 score)	307	36.8%	158 (51.5%)	
High (>60score)	478	57.2%	278 (18.6%)	

*= significant (*p* < 0.05).

Overall, 50 residents (6%) had a low level of social support (PSSS score < 37), 307 residents (36.8%) had a moderate level of social support (PSSS score ≥ 37 and ≤ 60), and 478 residents (57.2%) had a high level of social support (PSSS score > 60). Social support was negatively associated with PTSD. The prevalence of PTSD in respondents with a high level of social support (*n* = 278, 18.6%) was lower than that for respondents with a low level of social support (*n* = 31, 62%) or moderate level of social support (*n* = 158, 51.5%; *p* < 0.05) (Table 1).

**Figure 2:**
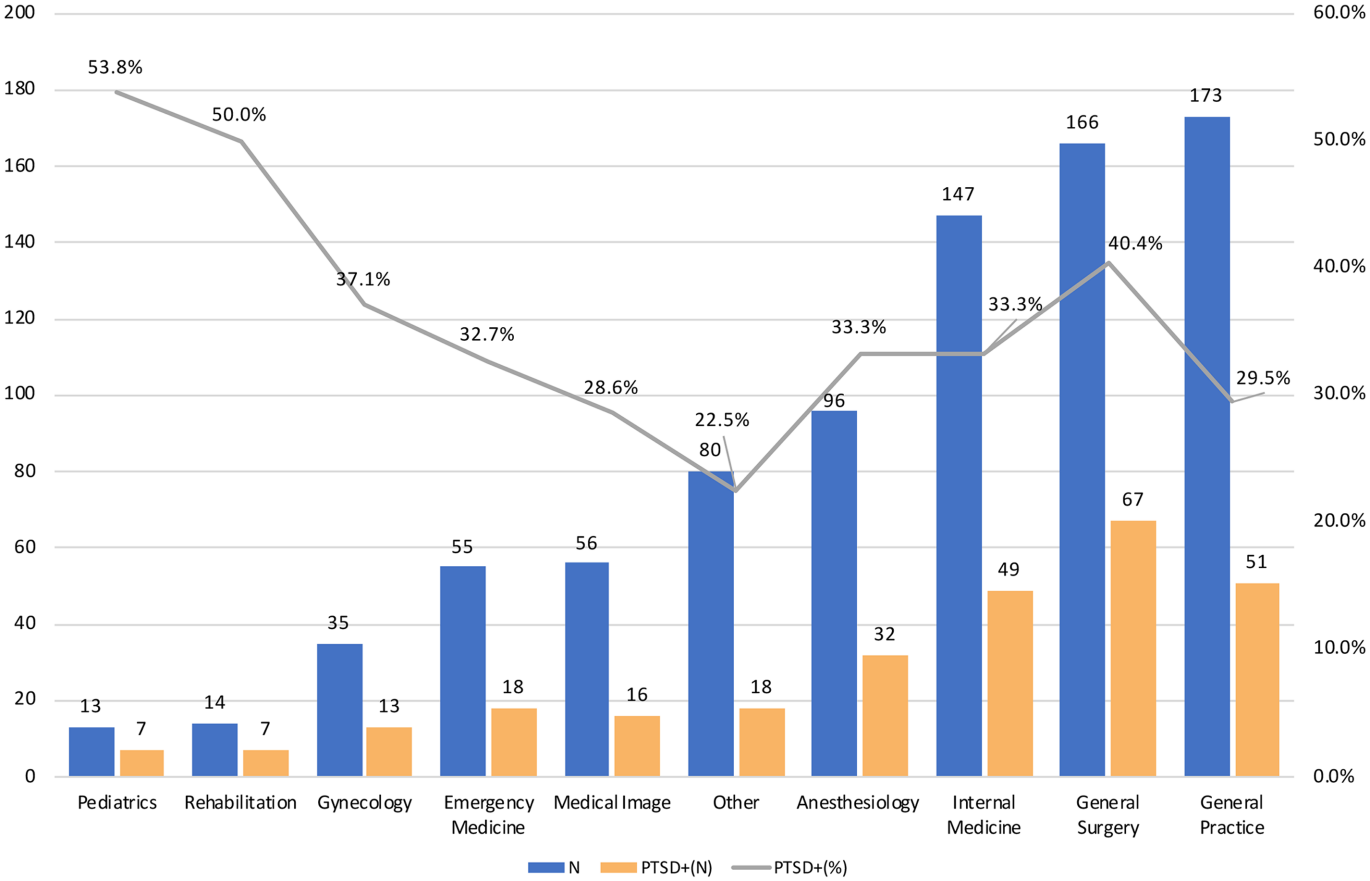
Comparison of screening positive for PTSD between medical specialties.

Figure 2 shows the prevalence of PTSD among different specialties. The specialties represented in the cohort were internal medicine (*n* = 147, 17.6%), surgery (*n* = 166, 19.9%), gynecology (*n* = 35, 4.2%), pediatrics (*n* = 13, 1.6%), general practice (*n* = 173, 20.7%), emergency medicine (*n* = 55, 6.6%), anesthesiology (*n* = 96, 11.5%), medical imaging (*n* = 56, 6.7%), rehabilitation (*n* = 14, 1.7%), and other (*n* = 80, 9.6%). The specialties with the lowest prevalence of PTSD were general practice (20.7%), surgery (19.91%), and internal medicine (17.6%). Except for the specialties of pediatrics and rehabilitation, which had a small number of respondents, the medical specialties with the highest proportion of individuals screening positive for PTSD were surgery (40.4%), gynecology (37.1%), internal medicine (33.3%), and anesthesiology (33.3%). Overall, 33.3% of the sample (*n* = 278) screened positive for PTSD.

Table 2 shows the multivariate analysis of potential modifiable and nonmodifiable risk factors for PTSD among all specialties. Of the surveyed demographic and occupational characteristics, being female (odds ratio (OR) = 0.601, *p* = 0.003), being aged 26–30 years (OR = 1.578, *p* = 0.018), and working an average of 50–60 h per week (OR = 1.789, *p* = 0.010), 60–70 h per week (OR = 2.725, *p* < 0.001), 70–80 h per week (OR = 2.014, *p* = 0.039), and > 80 h per week (OR = 4.365, *p* < 0.001) were significantly associated with a greater probability of having PTSD symptoms. There were no significant differences (*p* > 0.05) in the prevalence of PTSD among residents based on their education, medical specialty, marriage status, or monthly salary.

**Table 2:** Multivariate analysis of potential risk factors for screening PTSD positive.

Variables	OR	CI 95%		*p*
		Lower	Upper	
*Sex*				
Female	Ref			
Male	0.601	0.431	0.840	0.003*
*Age*				0.108
20–25	Ref			
26–30	1.578	1.081	2.305	0.018*
>30	1.949	0.919	4.131	0.082
*Education*				0.306
Undergraduate	Ref			
Graduate	0.852	0.566	1.283	0.443
Doctor	0.631	0.352	1.134	0.124
*Medical specialties*				0.332
Internal medicine	Ref			
Surgery	1.009	0.605	1.681	0.973
Gynecology	1.212	0.539	2.730	0.642
Pediatrics	2.824	0.848	9.407	0.091
General practice	0.927	0.541	1.589	0.784
Emergency medicine	0.880	0.438	1.766	0.719
Anesthesiology	1.016	0.559	1.848	0.958
Medical image	0.839	0.413	1.705	0.628
Rehabilitation	2.497	0.785	7.943	0.121
Other	0.597	0.309	1.153	0.125
*Medical practitioner or not*				
Yes	Ref			
No	0.858	0.573	1.285	0.457
*Marital status*				0.768
Single	Ref			
Married	0.833	0.508	1.364	0.467
*Average hours work per week*				0.000*
<50 h	Ref			
50-60 h	1.789	1.151	2.782	0.010*
60-70 h	2.725	1.663	4.464	0.000*
70-80 h	2.014	1.036	3.915	0.039*
>80 h	4.365	2.346	8.122	0.000*
*Monthly salary(¥)*				0.329
<5,000	Ref			
5,001–8,000	0.971	0.687	1.372	0.866
8,001–10,000	0.810	0.427	1.535	0.518
10,001–15,000	0.286	0.076	1.080	0.065
>15,000	0.302	0.032	2.877	0.298

*= significant (*p* < 0.05).

**Figure 3:**
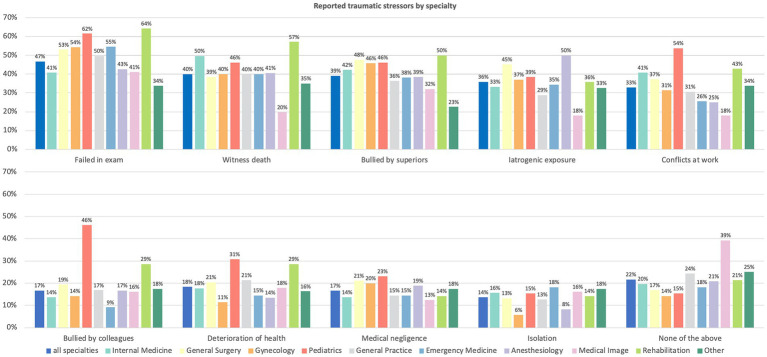
Reported traumatic stressors by specialty.

Overall, 654 (78%) residents reported experiencing at least one traumatic event. The most common traumatic stressors were “failing an examination” (47%), “witnessing death” (40%), and “bullying by superiors” (39%) (Figure 3). Traumatic stressors among specialties were compared and, in general, there were similar trends among them. In addition, traumatic stressors were related to the work content and context of the specialism. For example, the number of events of “witnessing death,” “conflicts at work,” and “iatrogenic exposure” in the specialty of medical imaging was lower than that for other specialties.

Multivariate analysis with adjustment for potential confounders showed that the medical profession-related traumatic events of “criticism/bullying by senior doctors” (OR = 2.269，*p* < 0.001), “criticism/bullying by other SRTP residents (disharmonious relationship)” (OR = 2.366，*p* < 0.001), “failing an important examination” (OR = 2.666, *p* < 0.001), and “medical isolation/separation” (OR = 2.105, *p* = 0.003) were risk factors for PTSD (Table 3).

**Table 3:** The multivariate analysis of PTSD screening positive respondents and medical-relevant traumatic events experiences.

Variables	OR	CI 95%		*p*
		Lower	Upper	
Criticism/bullying by superior doctors	2.269	1.581	3.256	0.000*
Criticism/bullying by other SRTP residents (disharmonious relationship)	2.366	1.487	3.765	0.000*
Conflict with patients/family members, be threatened or attacked	1.462	0.998	2.141	0.051
Iatrogenic exposure/infection	1.256	0.877	1.798	0.213
Patients died/deteriorated	1.379	0.968	1.966	0.075
Medical negligence and malpractice	1.077	0.672	1.726	0.759
Failure in important exam	2.666	1.875	3.791	0.000*
Medical-isolated or separated	2.105	1.298	3.415	0.003*
Yourself or hospital colleague fall in serious ill	0.927	0.596	1.442	0.738

*= significant (*p* < 0.05).

## 4. Discussion

A number of studies have confirmed the negative psychological impact of experiencing a disaster, suggesting that the subsequent risk of suffering from PTSD is substantial (19). The continuous tussle with unfavorable conditions related to the COVID-19 pandemic has increased the risk of HCWs suffering from PTSD and its symptoms. To clarify the characteristics of medical-related PTSD among SRTP residents in Shanghai during the COVID-19 pandemic, we conducted a cross-sectional survey and included 835 valid responses for analyses. We found that 654 residents (78.3%) had experienced at least one traumatic event, and that 278 residents (33.3%) had PTSD symptoms. Being 26–30 years of age, female, and having long working hours were identified as risk factors for PTSD, and perceived social support had a significant negative association with PTSD.

Overall, 33.3% of SRTP residents were found to have PTSD symptoms. This prevalence is significantly higher compared with that in research on PTSD among HCWs in the three previous outbreaks of coronavirus-related diseases (5). For example, Lin et al. (20) reported a PTSD prevalence of 21.7% among emergency-department staff after SARS 2003, whereas Zhang et al. (21) reported a PTSD prevalence of 12.4% among HCWs in high-risk areas during the COVID-19 pandemic. Studies have reported an increased risk of psychiatric disease and stress-related disorders among HCWs during the COVID-19 pandemic, as well as a high prevalence of PTSD symptomatology (22–24). Kheirallah et al. (25) reported a significant proportion of medical students from Jordan self-reporting an increased level of anxiety (49.2%) and depression (23.1%). Exposure to medical-related stressful events outside the range of normal human experience is part of the job for all HCWs, and the increased prevalence of PTSD in this group is not a new concept (especially during severe pandemics). However, what is an acceptable prevalence of PTSD for SRTP residents?

Among the demographic characteristics of SRTP residents, age, sex, and long working hours were associated with PTSD prevalence. Studies have demonstrated a higher level of work-related stress and “burnout” among female physicians compared with that experienced by male physicians (26, 27). In addition, the high prevalence of PTSD among HCWs is due to long working hours and work-related stress. We found the long working hours of residents to be associated with PTSD prevalence. Therefore, interventions to reduce working hours and, thus, improve physician wellbeing, are needed urgently.

Social support has been proposed to be the most efficacious way to alleviate the physical and emotional impacts of stressors (28). Social support has a critical role in the emotional, cognitive, and behavioral aspects of PTSD (29). Zalta and colleagues identified a lack of social support after trauma as a risk factor for PTSD, with a perceived lack of social support leading to a higher level of PTSD symptoms (30). A significant negative correlation between PTSD prevalence and perceived social support was demonstrated in the present study. Advice from friends, family, and significant others may enable subsequent behavioral changes that make everyday tasks more efficient or positive. Therefore, understanding, respecting, supporting, and empathizing with SRTP residents is fundamental in promoting their mental health in their medical careers.

Failing an examination, witnessing death, and bullying by superiors were the most commonly reported traumatic stressors among all SRTP residents. In general, there were similar trends of reported traumatic stressors between SRTP specialties. In addition, traumatic stressors were related to the work content and context of the specialism. For example, the prevalence of “witnessing death,” “conflicts at work,” and “iatrogenic exposure” in the specialty of medical imaging was lower than that in the other medical specialties. More than 45% of SRTP residents specializing in pediatrics answered “yes” to experiencing five traumatic stressors, indicating the high pressure and workload of pediatric medicine in China (31).It is not surprising that “failing an examination” was listed as the most common stressor among all SRTP residents. To ensure training quality, residents must complete the entire SRTP course and pass all examinations before they can apply for the exit examination (13). The latter is critically important, and those who do not pass it cannot graduate from the STRP and practice medicine in Shanghai. In addition, they receive only one more opportunity to take the examination after ≥6 months of training.

Isolation is an effective form of public-health management to prevent the spread of infectious diseases. However, isolation is associated with negative psychosocial effects, including depression, anxiety, anger, and PTSD. Samrah et al. (32) reported on isolated patients suffering from COVID-19 in Jordan; 44% reported symptoms of depression, and 21% were at high risk of major depressive disorder. HCWs (especially SRTP residents) may also develop PTSD as a result of isolation.

The present study had two main limitations. First, we focused on the occurrence of and risk factors for PTSD among STRP residents instead of clinical diagnoses and therapy. Therefore, the self-reported screening measures of PTSD and social support were applied in our study, but clinician-administered measures may have led to different results. Second, our cross-sectional research showed a strong bidirectional relationship between PTSD and social support, but we could not determine the direction of causality between social support and PTSD symptoms. Further research may be needed to ascertain if our findings can be replicated using longitudinal data.

## 5. Conclusion

The present findings demonstrate that there was a high prevalence of PTSD among SRTPs residents in Shanghai during the COVID-19 pandemic. The age 26–30 years old, female sex, and increased resident working hours were identified as risk factors for PTSD, while perceived social support was identified as a protective factor against PTSD. The present findings can be applied in SRTPs management for designing special interventions and protocols to protect the mental health of SRTPs residents.

## Data availability statement

Publicly available datasets were analyzed in this study. This data can be found here: the datasets presented in this article are not readily available because the data includes sensitive and private information. According to the ethical approval we cannot share it with the third party.

## Ethics statement

The studies involving humans were approved by the Department of Ethics Commission of Ruijin Hospital/Lu Wan Branch, School of Medicine, Shanghai Jiaotong University. The studies were conducted in accordance with the local legislation and institutional requirements. The participants provided their written informed consent to participate in this study.

## Author contributions

RH participated in conception, design of the work, data interpretation and analysis, drafting, and revision of the manuscript. CT, TL, LW, and CL participated in the acquisition and interpretation of data. JL and LC participated in the data analysis and revision of the draft. LC and SW made contributions to the concept and design of the study, acquisition of data, manuscript revision, and supervision. All authors approved the publication of this final version.
